# Neoadjuvant famitinib and camrelizumab, a new combined therapy allowing surgical resection of the primary site for anaplastic thyroid carcinoma

**DOI:** 10.1002/cnr2.1770

**Published:** 2022-12-19

**Authors:** Shuwen Yang, Dongmei Ji, Fen Xue, Tongzhen Chen, Yu Wang, Qinhai Ji

**Affiliations:** ^1^ Department of Head and Neck Surgery Fudan University Shanghai Cancer Center Shanghai China; ^2^ Medical Oncology Fudan University Shanghai Cancer Center Shanghai China; ^3^ Radiation Oncology Fudan University Shanghai Cancer Center Shanghai China; ^4^ Pathology Fudan University Shanghai Cancer Center Shanghai China

**Keywords:** anaplastic thyroid cancer, camrelizumab, famitinib, radiation therapy, surgery

## Abstract

**Background:**

Anaplastic thyroid cancer (ATC) is considered the most lethal thyroid cancer, with an overall 5‐year survival rate below 10%. The FDA approved a BRAF/MEK inhibitor combination for the treatment of patients with BRAF‐mutated ATC. However, effective therapeutic options for patients with wild‐type BRAF are lacking.

**Case:**

In our phase II study, patients having advanced/metastatic solid ATCs were treated with famitinib and camrelizumab, a combination therapy involving a multi‐targeted kinase inhibitor and an anti‐PD‐1 antibody. We report a case of a patient with locally advanced unresectable ATC who underwent this combination therapy, allowing us to perform complete surgical resection followed by post‐operative radiation therapy.

**Conclision:**

To the best of our knowledge, this is the first report describing the use of famitinib and camrelizumab as a neoadjuvant treatment for ATC with wild‐type BRAF. Clinical trial for a novel neoadjuvant approach for ATC are currently open for enrollment.

## 1 INTRODUCTION

Anaplastic thyroid cancer (ATC) is an extremely aggressive disease. It originates from follicular thyroid cells. It is the most dedifferentiated subtype of thyroid cancer and does not retain any biological characteristics of the follicular cells. Thyroid cancer stem cells can also play a role in the initiation and growth of ATCs. BRAF V600E mutation occurs in 25%–45% of the ATCs.^1^ As a permanently activated kinase, mutated BRAF V600E can phosphorylate its downstream targets such as MEK and ERK and is associated with aggressive features such as lymph node involvement, large tumor masses, or extrathyroidal metastases; leading to a poor prognosis.^2^, ^3^, ^4^

Anaplastic thyroid cancer has an overall survival of 6 months following diagnosis^5^ and accounts for more than half of all deaths related to thyroid cancers. Most of the patients with ATC show a poor response to systemic chemotherapy. Recent diagnostic methods such as individual mutation tests and in vitro assessment of patients ATC cells have made tailored treatments more feasible to improve the success rate of treatment and to avoid ineffective or harmful therapy. In differentiated thyroid cancer (DTC), copy number increases are present in the receptor tyrosine kinase genes (i.e., PDGFRα, PDGFRβ, PIK3Cb, VEGFR1, VEGFR2, PIK3Ca, EGFR, KIT, PDK1, and MET).^6^ An increased copy number is more common in ATC than in DTC and most of these genes play a decisive role in the carcinogenesis of ATC. Epigenetic alterations in thyroid carcinogenesis, which involve changes that drive poorly differentiated thyroid cancer and ATC, have attracted extensive attention. Cabanillas et al.^7^ and Wang et al.^1^ demonstrated the feasibility of complete surgical resection and locoregional disease control in patients with BRAF V600E‐variant tumors undergo surgical resection following BRAF‐directed neoadjuvant therapy. Based on the results of clinical trials, dabrafenib and trametinib have been approved by the FDA as feasible treatment options for patients having ATC with BRAFV600E‐variant and for those with locally advanced, metastatic, or unresectable ATC.

However, effective therapeutic options for patients with wild‐type BRAF are lacking. Immunotherapy with anti‐PD‐L1 antibodies, alone or in combination with BRAF inhibitors, has shown promising result for the treatment of ATC.^8^, ^9^ Several clinical studies have reported the use of camrelizumab (SHR‐1210); a humanized, high‐affinity, selective IgG4‐κ monoclonal anti‐PD‐1 antibody; which has shown good efficacy and acceptable safety in a wide range of solid tumors.^10^, ^11^, ^12^ Recently, therapeutic processes using multiple target agents have been explored and the results of these clinical trials have shown great promise in terms of overall survival rates as well as tumor shrinkage and progression‐free survival (PFS).^8^ In a phase II study, we explored the efficacy and safety of a combination therapy involving camrelizumab and famitinib. Camrelizumab is an anti‐PD‐1 inhibitor and famitinib, a multi‐target drug, is used to inhibit angiogenesis in the treatment of advanced ATC.^13^

We report a case of a patient with locally advanced, unresectable ATC who was treated with famitinib and camrelizumab, allowing us to perform complete surgical resection, followed by postoperative radiation therapy.

## 2 CASE DESCRIPTION

**FIGURE 1:**
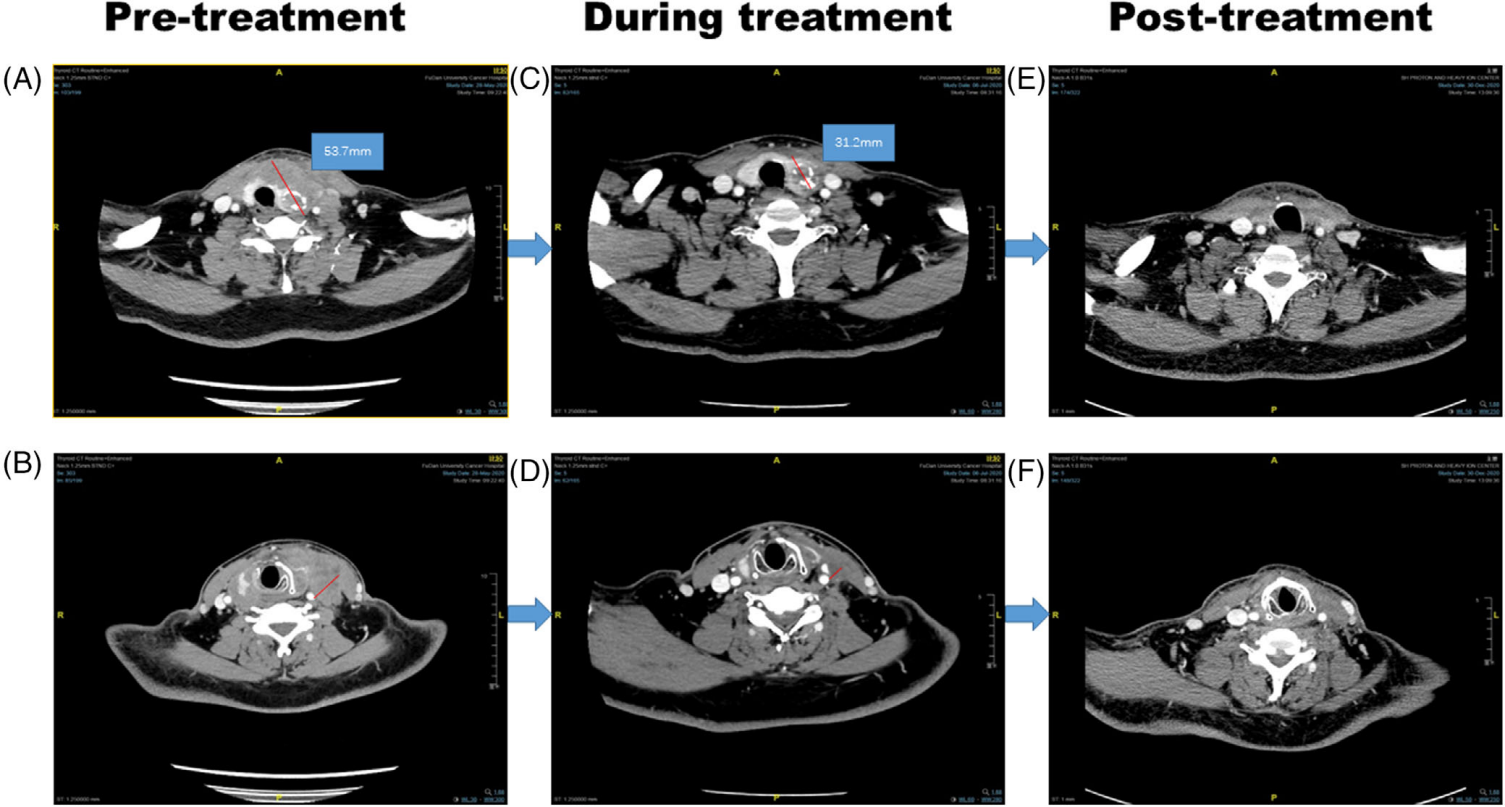
The effects of the therapy on the disease. Tumor size and cervical nodes size change from (A) and (B) baseline; (C) and (D) after three cycles of famitinib and camrelizumab treatment; (E) and (F) then after surgery and external radiation therapy.

**FIGURE 2:**
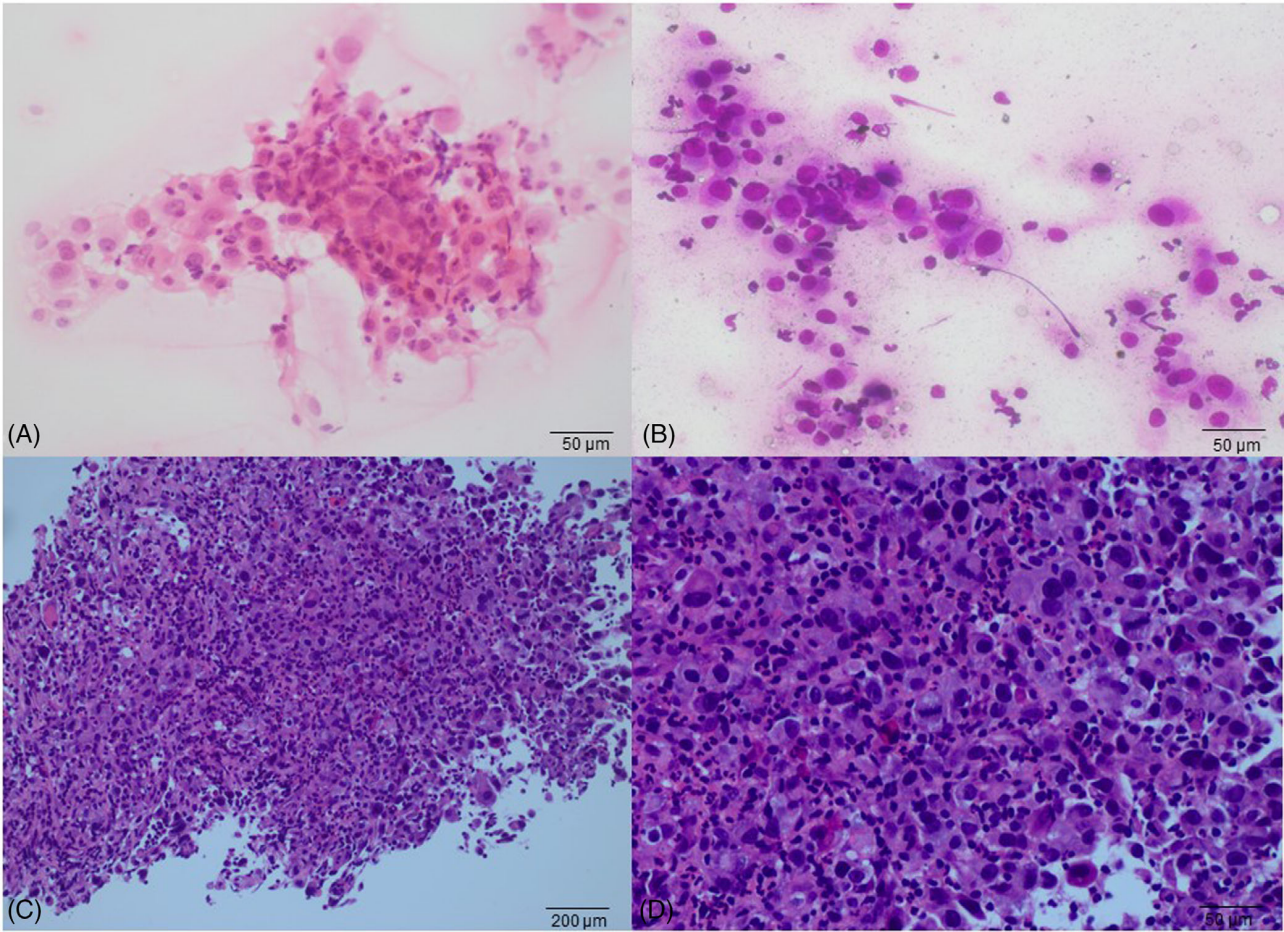
Core needle biopsy on left lobe: (A) and (B) the touch imprint cytology show the neoplastic tumor cells with obvious pleomorphism and nucleoli in some cells. (A) is hematoxylin and eosin histology section at 400× and (B) is Liu's stain at 400×. (C) the epithelioid tumor cells show solid growth cells which are obviously heteromorphic (hematoxylin and eosin histology section at 100×). (D) the nuclei of tumor are large, nucleoli are apparent and mitosis is easy to see (hematoxylin and eosin histology section at 400×).

**FIGURE 3:**
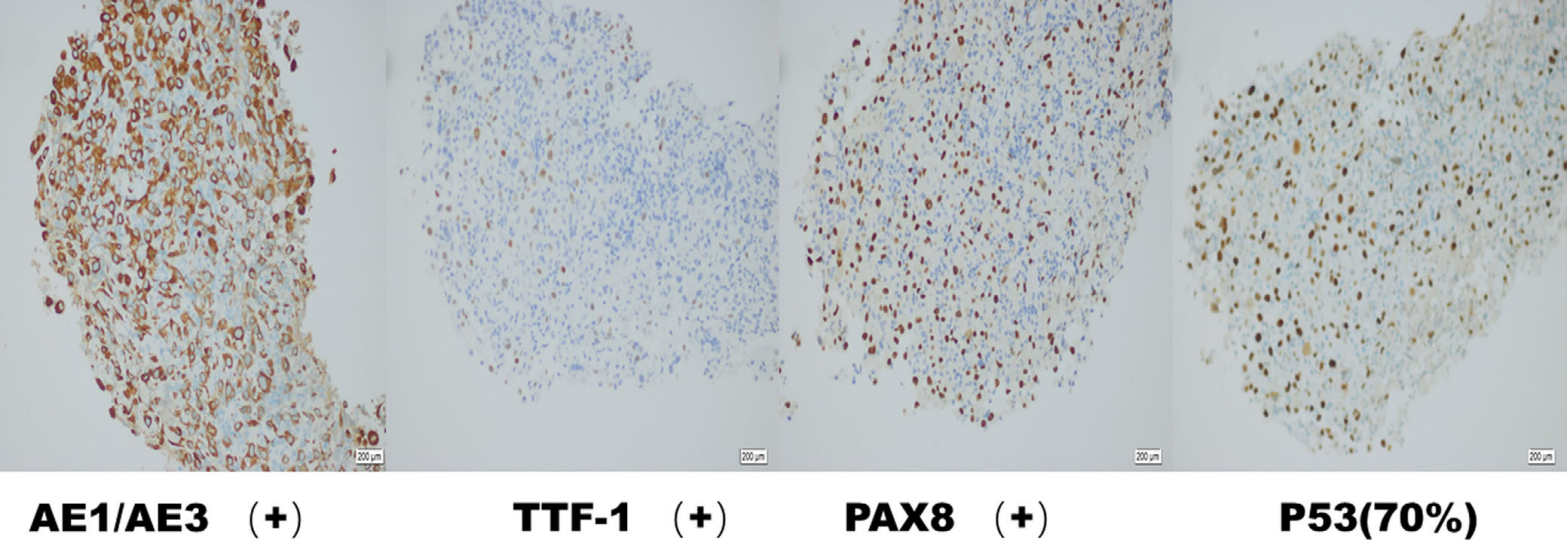
Immunohistochemically stained slides of core needle biopsy revealed the tumor cells to be positive for AE1/AE3, TTF‐1, PAX8, P53 (70%) at 200×.

A 51‐year‐old man inadvertently noticed a cervical mass that had been growing rapidly for 1 month. On May 26, 2020, the patient went to the outpatient department of Fudan University Shanghai Cancer Center. He had no dyspnea and was in good clinical condition with an Eastern Cooperative Oncology Group performance status score of 0. However, he complained of pain on touching the mass. Therefore, cervical ultrasound (US) and thyroid computed tomography (CT) were performed in May 2020. Imaging results showed calcified masses on both the lobes and isthmus of the thyroid, suggesting the possibility of thyroid carcinoma. The trachea was shifted to the right and invasion of the internal jugular vein and common carotid artery was observed (Figure 1A,B). Additionally, thyroid CT revealed multiple enlarged cervical nodes at levels II, III, and IV on the left side, each measuring approximately 20 mm. Core needle biopsy (CNB) of the prevailing thyroid nodule confirmed the diagnosis of ATC (Figure 2). Our center performs either immunohistochemistry and/or next‐generation sequencing (88‐gene somatic mutation analysis panel) as a standard of care for molecular testing of the tumor. NGS revealed three mutations: NRAS c.182A > T (variant allele frequency [VAF] 4.0%), TP53 c.742C > T (VAF 4.9%), and TERT C228T (VAF 3.5%). It was showed in immunohistochemically stained slides that tumor cells were positive for AE1/AE3, CK19, PAX8, P53 (70%), Ki67 (80%), TTF‐1, HBME‐1 (Figure 3). The cells were negative for thyroglobulin, desmin, and p63. Physical examination revealed a hard nodule sized 2–3 cm in the thyroid region and the trachea was displaced to the right. Abdominal US, brain CT, and chest CT detected no additional metastases. The patient was diagnosed with ATC (cT4bN1bM0, IVB according to the American Joint Committee on Cancer [AJCC] Cancer Staging Manual, 8th Edition) and the tumor was considered unresectable.

In June 2020, the patient was recruited for a clinical trial that combined multiple‐target kinase inhibitors and anti‐PD‐1 antibodies in patients with advanced thyroid cancer (NCT04521348). He was administered three cycles of famitinib (20 mg/qd) and camrelizumab (200 mg/q3w). The patient exhibited a good clinical status, but suffered from famitinib‐induced grade 3 hypertension, which was controlled with nifedipine and valsartan. Famitinib was discontinued for 3 days during the second cycle.

After three cycles of treatment, CT showed partial response (PR) of the lesion according to the Response Evaluation Criteria in Solid Tumors (version 1.1) (Figure 1C,D). The invaded internal jugular and common carotid arteries were separated. A multidisciplinary team for head and neck cancer offered the patient two main therapeutic options: surgical intervention or medical therapy. After discussion with the patient and his family members, the patient consented to total thyroidectomy and radical neck dissection, followed by radiotherapy.

**FIGURE 4:**
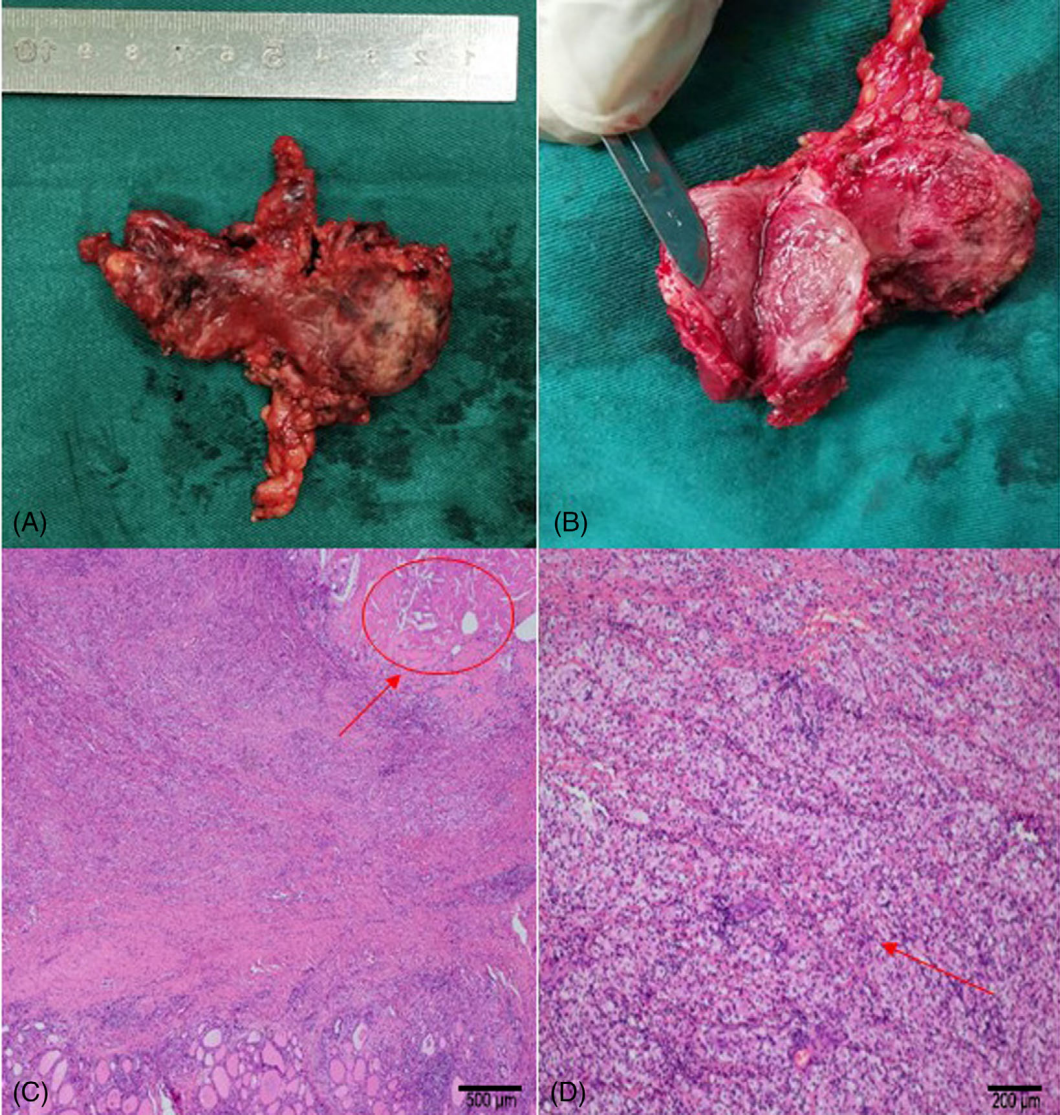
(A) and (B) show a surgical gross specimen; (C) and (D) show some foci poorly differentiated tumor nests were observed, with degeneration. Necrosis, desmoplasia, calcification, chronic inflammatory infiltration, and foamy histiocytes aggregates were found in surrounding areas, cholesterol crystals occasionally appeared (hematoxylin and eosin histology section at 40× and 100×).

Total thyroidectomy with removal of lymph node group VI and left radical neck dissection (groups II, III, IV, and V) were performed in July 2020. Postoperative pathological examination identified a 35‐mm (maximum diameter) lesion in the left thyroid lobe (Figure 4A,B) and some poorly differentiated tumor nests with degeneration. Necrosis, desmoplasia, calcification, chronic inflammatory infiltration, and foamy histiocyte aggregates were found in the surrounding areas. Cholesterol crystals were observed occasionally, which was consistent with the changes observed after treatment (Figure 4C,D). A 12‐mm (maximum diameter) lesion in the right thyroid lobe was diagnosed as papillary thyroid cancer. Necrosis, chronic inflammatory cell infiltration, foam cell aggregation, cholesterol crystals, and multinucleated giant cell reaction were observed in the anterior cervical mass, which was consistent with the changes after treatment. No definite residual carcinomas were observed. None of the 33 neck lymph nodes involved in neck dissection showed signs of metastatic carcinoma. Thus, the diagnosis of ATC was confirmed (ypT2N0M0, IVA according to AJCC Cancer Staging Manual, 8th Edition).

**FIGURE 5:**
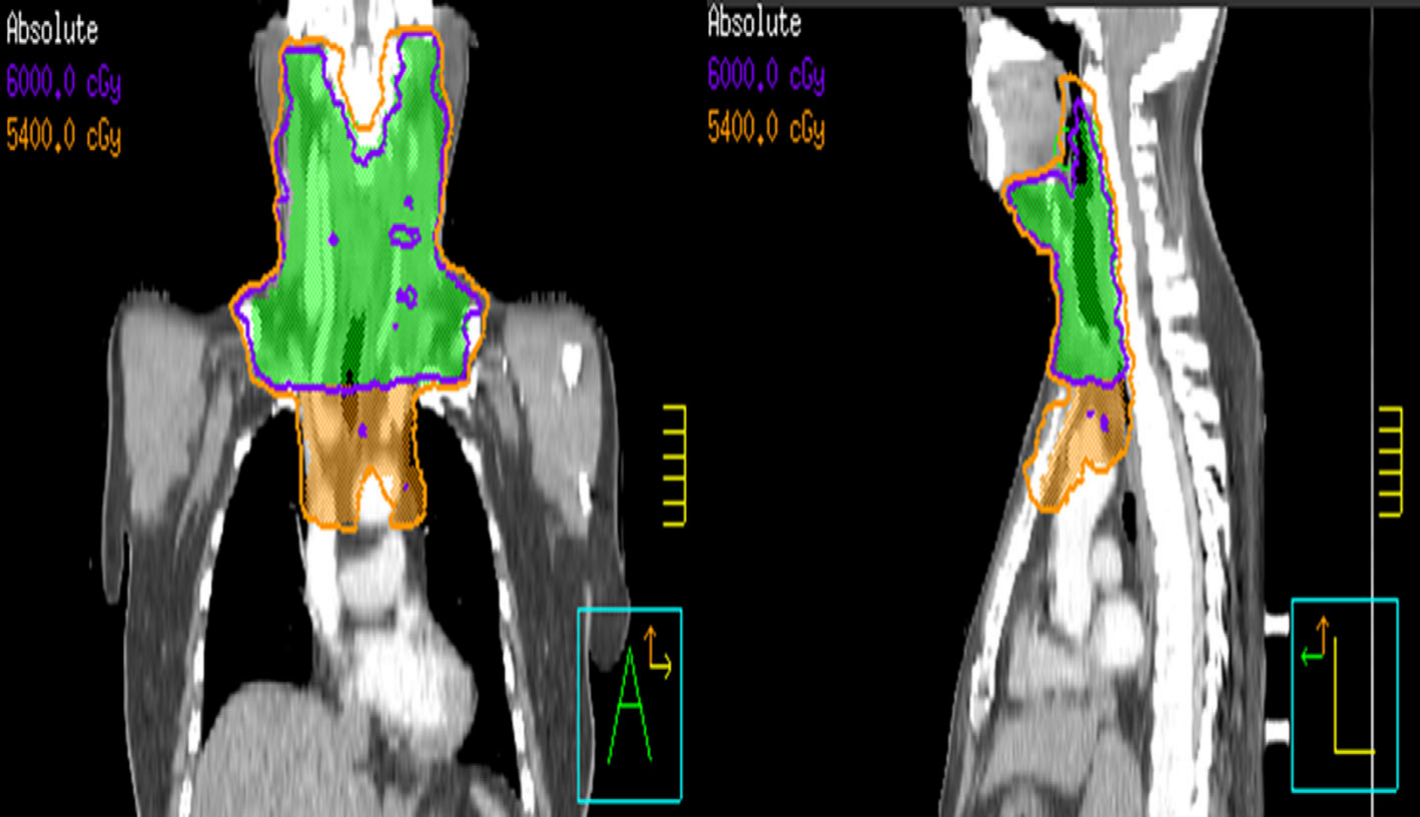
A coronal and a sagittal view of the post‐operative radiation planning CT scan. The blue and yellow lines represent the 60 Gy and 54 Gy isodose lines, respectively and the staining areas within these lines represent clinical targets specified at 60 and 54 Gy, respectively.

Postoperative therapy included L‐thyroxine (125 μg/d). Positron emission tomography CT showed no masses in the thyroid or neck lymph nodes and no distant metastasis. By August 2020, the patient had recovered well and received intensity‐modulated radiation therapy (IMRT) with a dose of 60 Gy (in 30 fractions) to the thyroid bed and cervical nodal regions and 54 Gy to the mediastinal nodes (Figure 5). Following radiation therapy, camrelizumab (200 mg/q3w) was administered for maintenance for 11 cycles. CT was performed to assess the response to camrelizumab in January 2021 (Figure 1E,F).

Surveys of patient‐reported outcomes were obtained approximately 24 months after the diagnosis. They showed a very good quality of life, including continuation of normal activities such as jogging.

## 3 DISCUSSION

Anaplastic thyroid cancer is considered the most lethal thyroid cancer, accounting for 38%–50% of all deaths related to thyroid cancers. The overall 5‐year survival rate for ATC is below 10%^14^ and it is still challenging to explore new treatment strategies to improve the survival of ATC patients. To the best of our knowledge, this is the first report describing the use of famitinib and camrelizumab as a neoadjuvant therapy for the management of ATC with wild‐type BRAF.

Anaplastic thyroid cancer often presents with severe symptoms including airway distress, hoarseness, dyspnea, and dysphagia due to a fast‐growing neck mass. A definitive diagnosis is usually made using fine‐needle aspiration biopsy and/or high‐resolution US. The patient in the present case underwent CNB of the prevailing thyroid nodule, which was diagnosed as ATC.

According to American Thyroid Association guidelines, a combination of surgery, chemotherapy, and radiotherapy should be used to manage and control local and metastatic ATCs.^15^ At our center, treatment of ATC usually involves surgical resection when feasible, followed by chemoradiotherapy. However, most of the patients with stage IVB ATC eventually develop distant metastasis and the prognosis for patients with stage IVC ATC is generally dismal despite multimodal therapy. Therefore, new ideas are needed for more effective control of tumors showing micrometastatic and distant diseases.

We attempted experimental therapy involving a combination of famitinib (a tyrosine kinase inhibitor [TKI]) and camrelizumab (an immune checkpoint inhibitor [ICI]). TKIs are currently used to treat aggressive thyroid cancers, as they induce clinical responses and stabilizes the disease. The role of TKIs in the treatment of thyroid cancer is limited to cases wherein progression continues after primary therapy (surgery and radioiodine therapy) and local ablation therapies. Careful evaluation of the risk/efficacy ratio is recommended in such cases. Testing each patient's sensitivity to different TKIs could pave the way for personalized treatment.^16^ A recent study evaluated the effectiveness of sunitinib in human cell lines obtained from ATCs (8305C and FB3). Sunitinib showed anti‐proliferative and pro‐apoptotic effects in endothelial and ATC cells, suggesting that it was effective in inhibiting ERK1/2 and the phosphorylation of Akt in activated endothelial and ATC cells in vivo and in vitro.^17^ In another study, a patient with ATC exhibited severe extensive residual disease and was treated with IMRT in combination with chemotherapy and sunitinib. For up to 18 months after the diagnosis, the patient exhibited complete response and no evidence of the disease.^18^ Another case report described a patient who was treated with sunitinib as a salvage treatment without previous systemic chemotherapy.^19^

Famitinib, a sunitinib analog, is a novel and highly potent multi‐target receptor tyrosine kinase inhibitor against c‐Kit, vascular endothelial growth factor receptor, and platelet‐derived growth factor receptor; with antitumor activity in the range of solid tumors.^20^, ^21^ Most of these molecular changes could be considered new diagnostic and prognostic molecular markers and therapeutic targets for ATC. Several studies have shown that famitinib‐treated tumors developed central necrosis or tumor vascularization, indicating that famitinib can effectively reduce tumor vascularity and induce tumor necrosis before tumor volume reduction. Simultaneously, broader synergistic mechanisms could play a role in preventing resistance to individual agents.^20^, ^21^, ^22^

In the present case, the primary thyroid lesion as well as cervical lymph nodes had shrunk following a combination therapy with a TKI and an ICI, which enabled us to perform complete surgical resection. After radiotherapy, the patient received camrelizumab for maintenance. Effective choices for systemic treatment of patients having ATC with wild‐type BRAF are still lacking. The present case report suggests that ATC with wild‐type BRAF could exhibit a substantial response to treatment with a combination of a TKI and an ICI. In a phase II cohort study, 42 patients were enrolled. The overall response rate was 19%, with complete response in 3 patients and partial response (PR) in 5 patients. This shows the reactivity of ATC to PD‐1 blockade.^9^ Furthermore, in 12 patients with progressive ATC, a combination of kinase inhibitors and pembrolizumab was evaluated. PR was observed in 42%, stable disease was observed in 33%, and progressive disease was observed in 25% of the patients.^23^ Several clinical studies have reported the efficacy of camrelizumab in a wide range of solid tumors. In China, 200 mg intravenous camrelizumab is recommended once every 2 weeks until intolerable toxicity or disease progression occurs. The derived neutrophils/(leukocytes minus neutrophils) ratio (dNLR) and lactate dehydrogenase (LDH) levels have been associated with the treatment outcomes of ICIs in lung cancer patients.^24^ Pre‐treatment lung immune prognostic index (LIPI) with dNLR below 3 and LDH level below the upper limit of normal (ULN) was associated with better treatment outcomes of ICIs. The patient in the present case had a dNLR of 1.2 (leukocytes: 7.1 × 10^9^/L and neutrophils: 3.9 × 10^9^/L) and an LDH level (180 U/L) below the ULN, which might be potential reasons for response to the combination therapy. We assumed that the combination of baseline dNLR and LDH might also be associated with treatment outcomes of ICIs in patients with ATC.

Although we cannot exclude the possibility that the patient might have responded to famitinib or camrelizumab monotherapy, the similarity of famitinib with sunitinib and the course of the disease suggest that the combination of famitinib and camrelizumab is more than just an addition of the two drugs. For patients already resistant to TKI therapy, the addition of pembrolizumab is partially effective with a PFS of only 2.96 months,^23^ suggesting that an upfront combination of famitinib and camrelizumab might be more effective than continuous medication.

After neoadjuvant therapy, the patient underwent major surgery including total thyroidectomy, bilateral central compartment dissection, and left neck dissection. The surgery successfully preserved critical functional organs such as the trachea, esophagus, parathyroids, and larynx/recurrent laryngeal nerves. Uruno et al. documented their clinical experience with neoadjuvant chemotherapy for ATC.^25^ In their trial, 16 patients were given paclitaxel with integration of multidisciplinary therapies including surgery and external beam radiation and they had a median survival of approximately 2 years. In another single‐institution cohort study,^26^ patients who received surgical resection following neoadjuvant BRAF‐directed therapy were alive at last follow‐up, with median follow‐up from diagnosis of 1.21 years (range 0.26–2.70 years). We speculate that this combination therapy may have prolonged survival among patients with ATC (24 months compared to a median survival of 5 months according to the literature^27^, ^28^).

In the neoadjuvant phase, toxicities related to famitinib were classified as grade I–II and the present study noted a lower incidence of side effects such as thrombocytopenia, leukocytopenia, neutropenia, and hypertension when compared with previous reports involving advanced solid malignancies refractory to standard treatment.^20^, ^22^ A possible reasons for this finding is that the patient had not undergone any previous treatment and was generally in good health.

In conclusion, ATC is associated with high mortality rates. Exploring new strategies is still needed to improve the survival of ATC patients. In this case, we chose a multidisciplinary method to treat this staging IVB ATC patient, which included the systemic treatment of famitinib and camrelizumab as the inducing therapy, and after the shrinkage of the tumor, surgery was performed and then adjuvant radiotherapy with subsequent maintenance immunotherapy was given. This approach is expected to be an effective therapeutic modality and should be explored prospectively in future clinical trials. Trials exploring combination immunotherapy and targeted therapy are currently ongoing (NCT04521348).

## AUTHOR CONTRIBUTIONS

**Shuwen Yang:** Conceptualization (equal); data curation (lead); formal analysis (equal); methodology (equal); software (equal); writing – original draft (lead). **Dongmei Ji:** Conceptualization (equal); data curation (equal); formal analysis (lead); resources (equal); writing – review and editing (equal). **Fen Xue:** Data curation (equal); software (equal); supervision (equal); validation (equal); visualization (lead). **Tongzhen Chen:** Data curation (equal); formal analysis (equal); visualization (lead). **Yu Wang:** Conceptualization (lead); investigation (lead); methodology (lead); project administration (lead); resources (equal); supervision (equal); writing – review and editing (equal). **Qinhai Ji:** Conceptualization (equal); data curation (equal); funding acquisition (equal); methodology (equal); project administration (lead); resources (lead); supervision (lead); validation (equal).

## CONFLICT OF INTEREST

The authors have stated explicitly that there are no conflicts of interest in connection with this article.

## ETHICS STATEMENT

All experiments were approved by Fudan University Shanghai Cancer Center institutional ethics committee (1910208‐17). Written informed consent was obtained from the patient for publication of this case report and any accompanying images.

## Data Availability

Not applicable.
